# Targeting *Hif1a* rescues cone degeneration and prevents subretinal neovascularization in a model of chronic hypoxia

**DOI:** 10.1186/s13024-018-0243-y

**Published:** 2018-03-07

**Authors:** Maya Barben, Christian Schori, Marijana Samardzija, Christian Grimm

**Affiliations:** 1 Laboratory for Retinal Cell Biology, Department of Ophthalmology, University Hospital Zurich, University of Zurich, Zurich, Switzerland; 2 Neuroscience Center Zurich (ZNZ), University of Zurich, Zurich, Switzerland; 3 Zurich Center for Integrative Human Physiology (ZIHP), University of Zurich, Zurich, Switzerland

**Keywords:** Retinal degeneration, Age-related macular degeneration, Cone photoreceptors, Hypoxia, HIF1, VHL, Mouse model

## Abstract

**Background:**

Degeneration of cone photoreceptors leads to loss of vision in patients suffering from age-related macular degeneration (AMD) and other cone dystrophies. Evidence, such as choroidal ischemia and decreased choroidal blood flow, implicates reduced tissue oxygenation in AMD pathology and suggests a role of the cellular response to hypoxia in disease onset and progression. Such a chronic hypoxic situation may promote several cellular responses including stabilization of hypoxia-inducible factors (HIFs).

**Methods:**

To investigate the consequence of a chronic activation of the molecular response to hypoxia in cones, von Hippel Lindau protein (VHL) was specifically ablated in cones of the all-cone *R91W;Nrl*^*-/-*^ mouse. Retinal function and morphology was evaluated by ERG and light microscopy, while differential gene expression was tested by real-time PCR. Retinal vasculature was analyzed by immunostainings and fluorescein angiography. Two-way ANOVA with Šídák’s multiple comparison test was performed for statistical analysis.

**Results:**

Cone-specific ablation of *Vhl* resulted in stabilization and activation of hypoxia-inducible factor 1A (HIF1A) which led to increased expression of genes associated with hypoxia and retinal stress. Our data demonstrate severe cone degeneration and pathologic vessel growth, features that are central to AMD pathology. Subretinal neovascularization was accompanied by vascular leakage and infiltration of microglia cells. Interestingly, we observed increased expression of tissue inhibitor of metalloproteinase 3 (*Timp3*) during the aging process, a gene associated with AMD and Bruch’s membrane integrity. Additional deletion of *Hif1a* protected cone cells, prevented pathological vessel growth and preserved vision.

**Conclusions:**

Our data provide evidence for a HIF1A-mediated mechanism leading to pathological vessel growth and cone degeneration in response to a chronic hypoxia-like situation. Consequently, our results identify HIF1A as a potential therapeutic target to rescue hypoxia-related vision loss in patients.

## Background

Age-related macular degeneration (AMD) is the leading cause of visual impairment in the elderly population in industrialized nations [1–3]. Due to the degeneration of photoreceptors in the cone-rich macula and/or the ingrowth of blood vessels, patients suffering from AMD lose central, high acuity vision [4–6]. While there is no therapy available for geographic atrophy (dry AMD), the neovascular (wet) form of AMD is treated by vascular endothelial growth factor (VEGF)-targeting therapies to slow disease progression [7–9]. Choroidal neovascularization (CNV) defines the classic form of neovascular AMD and is characterized by the ingrowth of blood vessels from the choroid to the subretinal space [6, 10]. Retinal angiomatous proliferation (RAP) has been described as an additional, distinct form of neovascular AMD [11]. In RAP, also known as deep retinal vascular anomalous complexes, vessels originate not from the choroid but from the deep retinal plexus in the inner retina and extend into the photoreceptor layer and the subretinal space [12, 13].

AMD is a multifactorial disease. Besides genetic and environmental risk factors [5, 14, 15], tissue hypoxia and changes in retinal blood flow have been implicated in its etiology [16–20]. Oxygen supply to photoreceptors in the eyes of elderly people may be impaired due to an age-dependent reduction of choroidal blood flow [21, 22] and accumulation of drusen [23]. Choroidal ischemia in dry AMD [24, 25] and decreased choroidal blood volume in AMD [26] further support the hypothesis that hypoxia might be implicated in disease development and progression. The retina is considered as one of the most metabolically active tissues and is therefore highly vulnerable to changes in oxygen tension [17, 27]. In conditions of reduced oxygen supply (hypoxia), molecular responses are activated with hypoxia-inducible factor 1 (HIF1) playing a key regulatory role for adapting the cell/tissue to the new condition. Heterodimeric HIF1 proteins are composed of an oxygen-labile α-subunit and a constitutively expressed β-subunit [28]. Under normoxic conditions, HIF1A is hydroxylated by prolyl hydroxylase domain (PHD) proteins. This promotes the interaction with the von Hippel-Lindau (VHL) ubiquitin E3 ligase complex leading to ubiquitination and rapid degradation of hydroxylated HIF1A by proteasomes. Under hypoxic conditions, hydroxylation of HIF1A is reduced. Hence, HIF1A accumulates, enters the nucleus and drives transcription of a multitude of target genes [29–31].

In this study, we investigated the consequences of a chronic hypoxia-like response triggered in cone photoreceptors to elucidate the mechanisms of cell death in cone degenerative diseases such as AMD. To this end, we used *R91W;Nrl*^*-/-*^ double-mutant mice which express only cone photoreceptors in a well-layered, functional retina [32]. To induce the hypoxia-like response, we ablated the VHL protein specifically from cones using the Cre-loxP system. We analyzed the effects of the cone-specific activation of the hypoxic response and validated the contribution of HIF1A to the resulting retinal pathology.

## Methods

### Mice

All experimental procedures were performed according to ‘The Association for Research in Vision and Ophthalmology’ statement on animal use in ophthalmic and vision research and the regulation of the veterinary authorities of Kanton Zurich, Switzerland. *R91W;Nrl*^*-/-*^ mice were generated by crossing *Rpe65*^*R91W*^ (R91W) [33] to *Nrl*^*-/-*^ mice [34], and were described recently [32]. *BPCre;R91W;Nrl*^*-/-*^*;Vhl*^*f/f*^ (=*cone*^*ΔVhl*^) mice were generated by breeding *R91W;Nrl*^*-/-*^ mice to *Vhl*^*f/f*^ mice [35] and mice expressing the *Cre* recombinase under the transcriptional control of the blue cone opsin (BP) promoter [36]. To generate *BPCre;R91W;Nrl*^*-/-*^*;Vhl*^*f/f*^*;Hif1a*^*f/f*^ (*=cone*^*ΔVhlHif1a*^) mice, *BPCre;R91W;Nrl*^*-/-*^*;Vhl*^*f/f*^ were bred to *Hif1a*^*f/f*^ mice [37]. *R91W;Nrl*^*-/-*^*;Vhl*^*f/f*^ and *R91W;Nrl*^*-/-*^*;Vhl*^*f/f*^*;Hif1a*^*f/f*^ littermates without *Cre* recombinase served as respective control mice (=*ctrl*). Genotyping was performed by PCR using DNA isolated from ear clips and primer pairs as described previously [38, 39]. Presence of *BPCre* was tested using the following primer pair: forward (5’-GGACATGTTCAGGGATCGCCAGGCG-3’) and reverse (5’-GCATAACCAGTGAAACAGCATTGCTG-3’). The amplification reaction resulted in a 268 bp fragment in the presence of the transgene. To test for deletion of floxed sequences, genomic DNA was isolated from retinal tissues and tested by PCR using appropriate primer pairs as described [38, 39]. *129S6* (Taconic, Ejby, Denmark) mice were used as wild-type controls. To test expression of the *Cre* recombinase *BPCre;R91W;Nrl*^*-/-*^ mice were bred to a ZsGreen reporter line (*Ai6* mice, *Gt(ROSA)26Sor*^*tm6(CAG-ZsGreen1)Hze*^, [40]). Mice of both sexes were used for experiments and were housed at the animal facility of the University Zurich under a 14 h : 10 h light/dark cycle with lights on at 6 am and lights off at 8 pm. Food and water were provided *ad libitum*.

### Morphology/quantification

To evaluate retinal morphology, eyes were enucleated and fixed in 2.5% glutaraldehyde in cacodylate buffer (pH 7.2, 0.1 M), according to the previously described procedure [41]. By cutting through the optic nerve head, nasal and temporal halves of the eyecups were separated and embedded in epon plastic. Semi-thin cross sections (0.5 μm) were counterstained with toluidine blue and analyzed by light microscopy (Axioplan; Zeiss, Jena, Germany). Thickness of the outer nuclear layer was measured at indicated distances from the optic nerve head using the Adobe Photoshop CS6 ruler tool (Adobe Systems, Inc., San Jose, CA, USA) on reconstructed retinal panorama images.

### Immunofluorescence

After euthanasia, eyes were marked nasally, enucleated and fixed in 4% paraformaldehyde (PFA) in phosphate buffer for 1 h at 4°C. Cornea and lens were removed and the dissected eyecups postfixed for 2 h in 4% PFA. After immersion in 30% sucrose (in PBS 0.1 M) the eyecups were embedded in tissue freezing medium (O.C.T., Leica Biosystems Nussloch GmbH, Nussloch, Germany), frozen in a 2-methylbutane bath cooled by liquid nitrogen and stored at -80°C. Cryosections (12 μm) were blocked (3% normal goat serum (Sigma-Aldrich, St. Louis, MO, USA), 0.3% Triton X-100 (Sigma) in PBS) and incubated with the following primary antibodies overnight at 4°C: Isolectin GS-IB_4_-Alexa594 from *Griffonia simplicifolia* (1:300, I21413; Thermo Fisher Scientific, Waltham, MA, USA), rabbit anti-allograft inflammatory factor 1 (alias IBA1, 1:1000, 019-19741; Wako, Neuss, Germany), rabbit anti-albumin (ALB, 1:500, RARaAlb; Nordic Immunology, Tilburg, Netherlands). Sections were washed with PBS, incubated with secondary antibodies (Cy3-labeled, Jackson ImmunoResearch Laboratories, Westgrove, PA, USA) for 1 h at room temperature (RT), counterstained with DAPI (4',6-Diamidine-2'-phenylindole dihydrochloride, Roche, Basel, Switzerland) and analyzed by fluorescence microscopy (Axioplan; Zeiss).

### Analysis of retinal vasculature in whole mounted retinas

Eyes were isolated and incubated for 5 to 10 minutes in 2% PFA in PBS as described recently [42]. After removal of cornea and lens, the retina was dissected and flat-mounted in PBS. After postfixation in 4% PFA for 1 h at RT, flat-mounts were blocked (3% normal goat serum, 0.3% Triton X-100 in PBS, 1 h) and incubated with isolectin GS-IB_4_-Alexa594 (1:300, Thermo Fisher Scientific) at 4°C overnight. Retinas were washed in PBS, mounted on glass slides and analyzed by fluorescence microscopy (Axioplan/ApoTome; Zeiss). Blood vessels were reconstructed in three dimensions using Imaris software (Versions 7.7.2/8.3.0, Bitplane AG, Zurich, Switzerland). For better recognition and distinction of the vascular plexi, the z-value of the z-stacks was increased five times.

### RNA isolation and semi-quantitative real-time PCR

Retinas were isolated through a slit in the cornea, frozen in liquid nitrogen and stored at -80°C. RNA was extracted using an RNA isolation kit (RNeasy; Qiagen, Hilden, Germany) including a DNAse treatment. 1 μg of RNA, oligo (dT) and M-MLV reverse transcriptase (Promega, Fitchburg, WI, USA) were used to prepare cDNA. To analyze gene expression by real-time PCR, 10 ng of cDNA template was amplified using a PCR polymerase ready mix (LightCycler 480 SYBR Green I Master, Roche Diagnostics, Rotkreuz, Switzerland), specific primer pairs (Table 1) and a thermocycler (LightCycler 480, Roche Diagnostics). Expression levels were normalized to β-actin (*Actb*) and relative expression was calculated using the comparative threshold cycle method (∆∆C_T_) of the LightCycler480 software (Roche Diagnostics). At least 3 mice per strain and time point were used.

**Table 1:** Primers used for real-time PCR

Gene	Forward (5’-3’)	Reverse (5’-3’)	Product (bp)
*Actb*	CAACGGCTCCGGCATGTGC	CTCTTGCTCTGGGCCTCG	153
*Adm*	TCCTGGTTTCTCGGCTTCTC	ATTCTGTGGCGATGCTCTGA	133
*Bnip3*	CCTGTCGCAGTTGGGTTC	GAAGTGCAGTTCTACCCAGGAG	93
*Casp1*	GGCAGGAATTCTGGAGCTTCAA	GTCAGTCCTGGAAATGTGCC	138
*Fgf2*	TGTGTCTATCAAGGGAGTGTGTGC	ACCAACTGGAGTATTTCCGTGACCG	158
*Gfap*	CCACCAAACTGGCTGATGTCTAC	TTCTCTCCAAATCCACACGAGC	240
*Glut1*	CAGTGTATCCTGTTGCCCTTCTG	GCCGACCCTCTTCTTTCATCTC	151
*Pdgfb*	GCTGCTGCAATAACCGCAAT	GTGGTCCTCCAAGGTCACTG	131
*Pdgfrb*	CTTGCCCTTCAAAGTGGTGG	CCAGGTGGAGTCGTAAGGC	199
*Egln1*	GCAGCATGGACGACCTGAT	CAACGTGACGGACATAGCCT	123
*Sema3f*	CGTCGCGCACAGGATTA	GGAAAATGGCTGCATCGGTA	166
*Timp3*	GCCTCAAGCTAGAAGTCAACAAA	TGTACATCTTGCCTTCATACACG	69
*Vegf*	ACTTGTGTTGGGAGGAGGATGTC	AATGGGTTTGTCGTGTTTCTGG	171
*vWF*	CCCTGGACAACTTGACAGCAG	ACAAGCAGGCAGATCTCATACC	192

### Protein isolation and Western blotting

Proteins were isolated by homogenizing the retinas in ice-cold Tris-HCl (100 mM, pH 7.5) using ultrasound at 4°C. Protein concentrations were determined spectrophotometrically by using Bradford reagent (Bio-Rad, Hercules, CA, USA). SDS-PAGE and Western blotting were performed as described [41] and proteins detected by rabbit anti-HIF1A (1:2000, NB100-479, Novus Biologicals, Littleton, CO, USA) and mouse anti-β-actin (1:10’000, A5441, Sigma-Aldrich) antibodies. HRP-conjugated secondary antibodies were applied for 1 h at RT and signals were visualized using Western lightning chemiluminescence reagent (PerkinElmer, Waltham, MA, USA) and X-ray films.

### Electroretinography (ERG)

Mice were dark-adapted overnight and pupils were dilated under dim red light with 1% cyclogyl (Alcon Switzerland SA, Rotkreuz, Switzerland) and 5% neosynephrin-POS (Ursapharm Schweiz GmbH, Roggwil, Switzerland) 30 minutes prior to recording. Mice were anesthetized with a subcutaneous injection of ketamine (85 mg/kg, Pfizer PFE Switzerland GmbH, Zurich, Switzerland) and xylazine (Rompun 2%, 4mg/kg, Bayer, Leverkusen, Germany). To keep the cornea moist, a drop of mydriaticum dispersa (Omnivision AG, Neuhausen, Switzerland) was applied to each eye. Low background illumination for 5 min was used for light adaptation. Electroretinograms were recorded simultaneously from both eyes with an LKC UTAS Bigshot unit (LKC Technologies, Inc. Gaithersburg, MD, USA) as described [32]. Flashes of 8 different light intensities ranging from -10 to 25 dB (0.25–790.5694 cd*s/m^2^) were applied under photopic conditions. 10 responses were averaged per light intensity. Traces from n≥4 mice were averaged for each light intensity.

### Fundus imaging and fluorescein angiography

Fundus photographs were taken with a Micron IV system (Phoenix Research Labs, Pleasanton, CA, USA). The pupils were dilated and the mice were anesthetized as described above. Methocel 2% (Omnivision) was applied to lubricate the eyes. Fluorescein images were captured 1-5 minutes after intraperitoneal injection of 20 μL of 2% fluorescein solution (Akorn, Lake Forest, IL, USA).

### Experimental design and statistical analysis

Two-way ANOVA with Šídák’s multiple comparison test was performed using GraphPad Prism (version 7.02, GraphPad Software, San Diego, CA, USA) for statistical analysis of gene expression levels and ERG traces. All data are shown as means ± SD.

## Results

### Cone-specific inactivation of *Vhl* increased HIF1A target gene expression and induced progressive cone degeneration

**Fig. 1:**
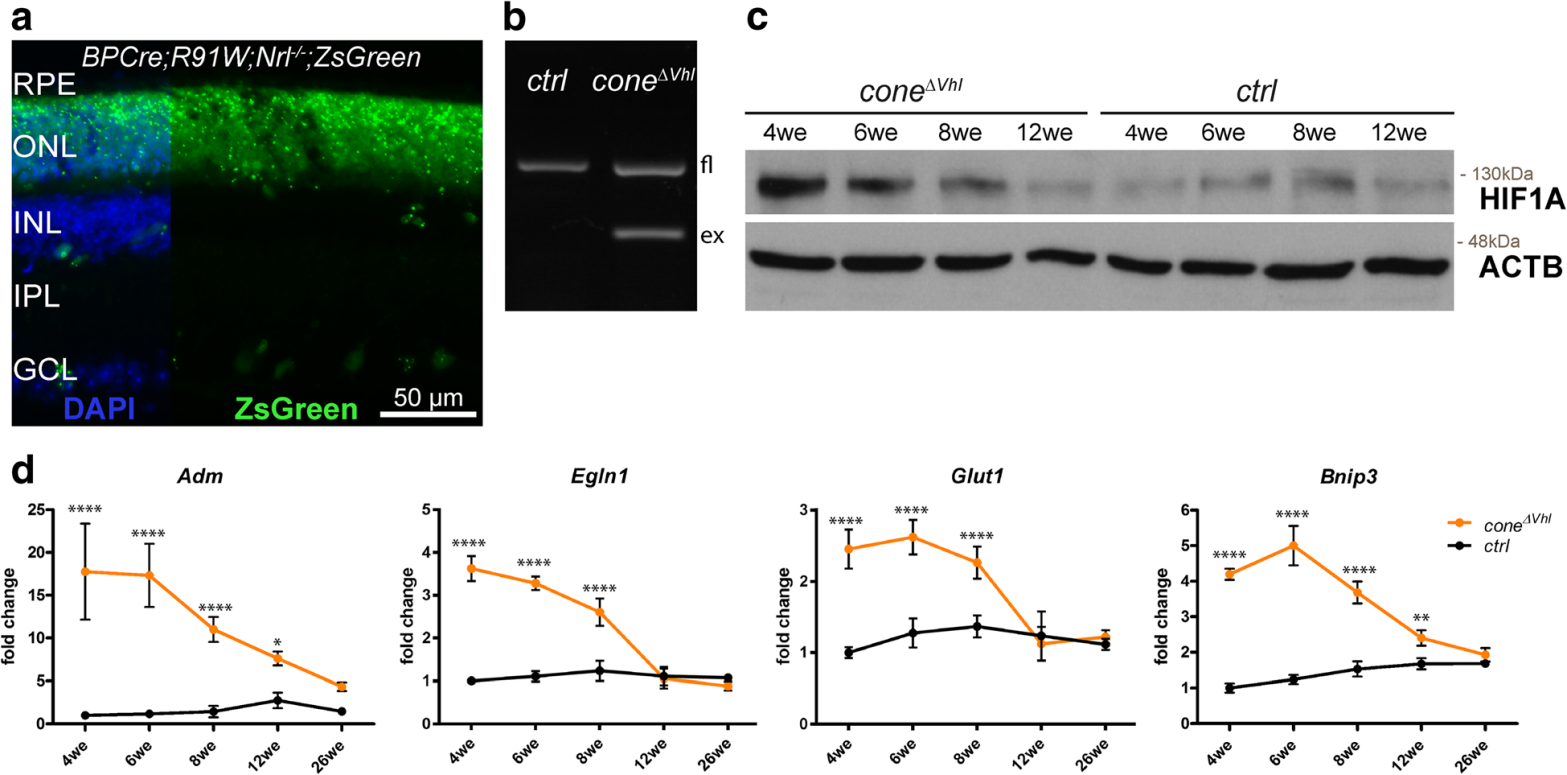
Knockdown of *Vhl* in cones of the all-cone mouse (*cone*^*ΔVhl*^). **a** Expression of *Cre* recombinase in *BPCre;R91W;Nrl*^*-/-*^*;ZsGreen* mice. Green fluorescence (ZsGreen) indicates *Cre* activity. RPE: retinal pigment epithelium, ONL: outer nuclear layer, INL: inner nuclear layer, IPL: inner plexiform layer, GCL: ganglion cell layer. Scale bar 50 μm. **b** PCR of genomic DNA from retinas of 4-week-old *ctrl* and *cone*^*ΔVhl*^ mice. Floxed *Vhl* (fl) is detected at 460 bp and *Cre*-mediated excision (ex) results in a 260 bp fragment. **c** Western blot analysis of HIF1A in 4, 6, 8 and 12-week-old *cone*^*ΔVhl*^ and *ctrl* (littermates without *Cre* recombinase) mice. ACTB served as loading control. **d** Relative expression of hypoxic target genes in retinas of *ctrl* (black) and *cone*^*ΔVhl*^ (orange) mice. Expression levels were normalized to beta-actin (*Actb*) and calculated relatively to 4-week-old *ctrl* mice, which were set to 1. Shown are means ± SD. Two-way ANOVA with Šídák’s multiple comparison test was used for statistical analysis (*Adm*: ****p<0.0001, *p=0.0306; *Egln1*: ****p<0.0001; *Glut1*: ****p<0.0001; *Bnip3*: ****p<0.0001, **p=0.0043; n≥3 per time point). *Adm*: Adrenomedullin, *Egln1*: egl-9 family hypoxia-inducible factor 1, *Glut1*: Glucose transporter 1, *Bnip3*: BCL2/adenovirus E1B interacting protein 3. *Cre*-negative littermates were used as *ctrl* mice in all experiments unless otherwise indicated

Photoreceptors in the all-cone *R91W;Nrl*^*-/-*^ mouse are predominantly blue-light sensitive S-cones [32, 34]. Therefore, we used *BP-Cre* transgenic mice, which express functional *Cre* recombinase under the transcriptional control of the blue cone opsin promoter (BP, [36]) to delete floxed sequences from cones in the all-cone mice. *BPCre;R91W;Nrl*^*-/-*^*;ZsGreen* reporter mice verified that *Cre* expression was uniform over the entire retina and restricted almost exclusively to the majority of cones in the ONL (Fig. 1a). Green fluorescence of the activated reporter protein was only occasionally detected in few cells of the inner retina. We validated *Vhl* excision in the *BPCre;R91W;Nrl*^*-/-*^*;Vhl*^*f/f*^ (=*cone*^*ΔVhl*^) mouse line by analyzing genomic DNA isolated from retinal tissues of 4-week-old *cone*^*ΔVhl*^ mice. The detection of a PCR product corresponding to the excised fragment suggested a successful *Cre*-mediated deletion of *Vhl* in retinas of *cone*^*ΔVhl*^ but not of *ctrl* mice (=*R91W;Nrl*^*-/-*^*;Vhl*^*f/f*^, Fig. 1b). Genomic deletion of *Vhl* led to the accumulation of HIF1A protein (Fig. 1c) and to increased transcript levels of known hypoxic target genes such as adrenomedullin (*Adm*), egl-9 family hypoxia-inducible factor 1 (*Egln1*, also known as *Phd2*), glucose transporter 1 (*Glut1*) and BCL2/adenovirus E1B 19-kDa interacting protein 3 (*Bnip3*) in normoxic *cone*^*ΔVhl*^ mice (Fig. 1d). This suggested that cells, presumably cones, activated a hypoxia-like response in retinas of *cone*^*ΔVhl*^ mice.

**Fig. 2:**
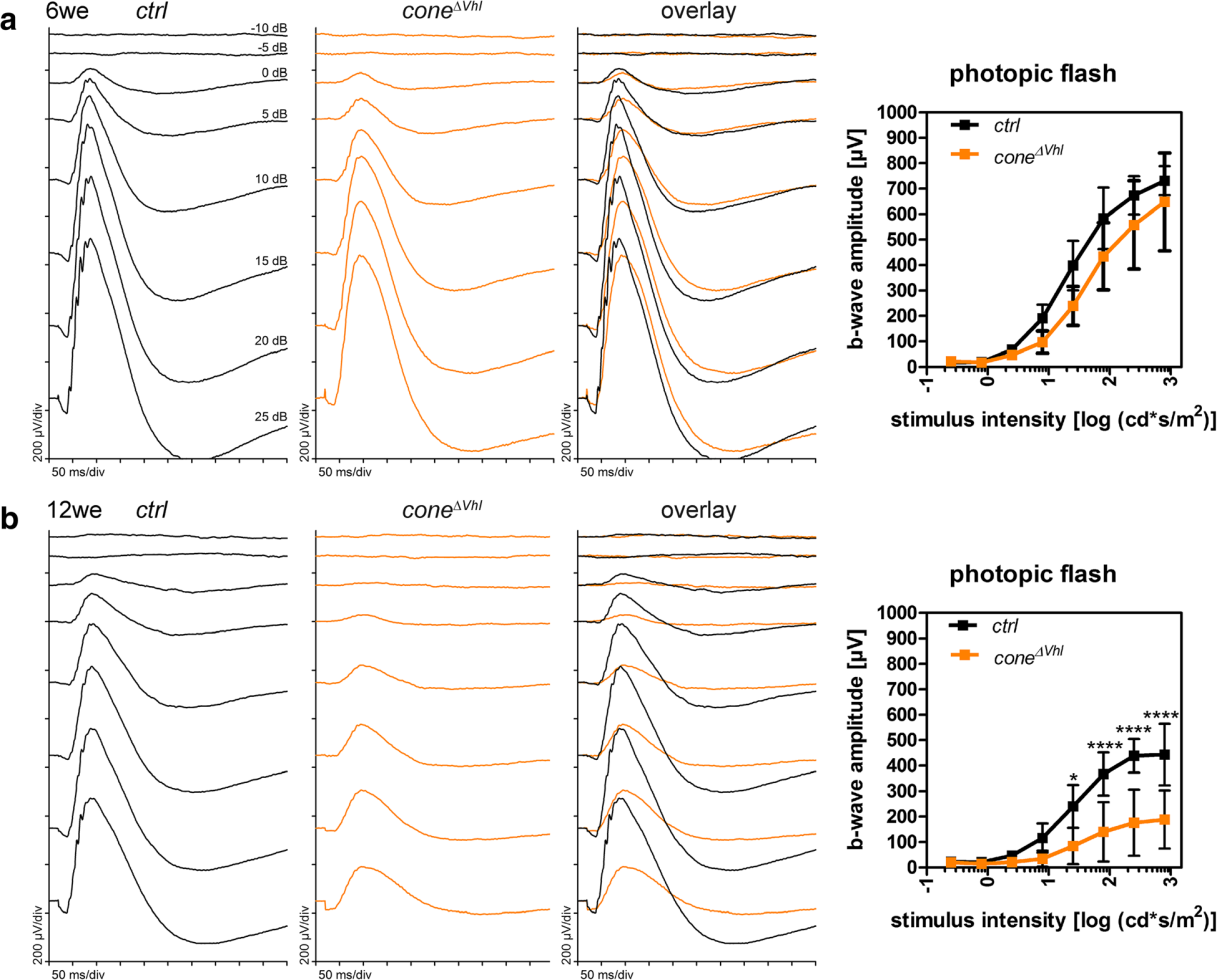
Reduced retinal function in aged *cone*^*ΔVhl*^ mice. **a** Averaged traces of single-flash photopic ERGs and b-wave amplitudes showed no significant differences in *cone*^*ΔVhl*^ mice (orange) compared to *ctrl* mice (black) at 6 weeks of age. **b** At 12 weeks of age, photopic b-wave amplitudes were strongly reduced in *cone*^*ΔVhl*^ mice (orange) as compared to *ctrl* mice (black). b-wave amplitudes are shown as means ± SD. Two-way ANOVA with Šídák’s multiple comparison test was used for statistical analysis (6we *ctrl* and *cone*^*ΔVhl*^ *n*=5, 12we *ctrl n*=6 and *cone*^*ΔVhl*^ *n*=4, *p=0.0115, ****p<0.0001)

**Fig. 3:**
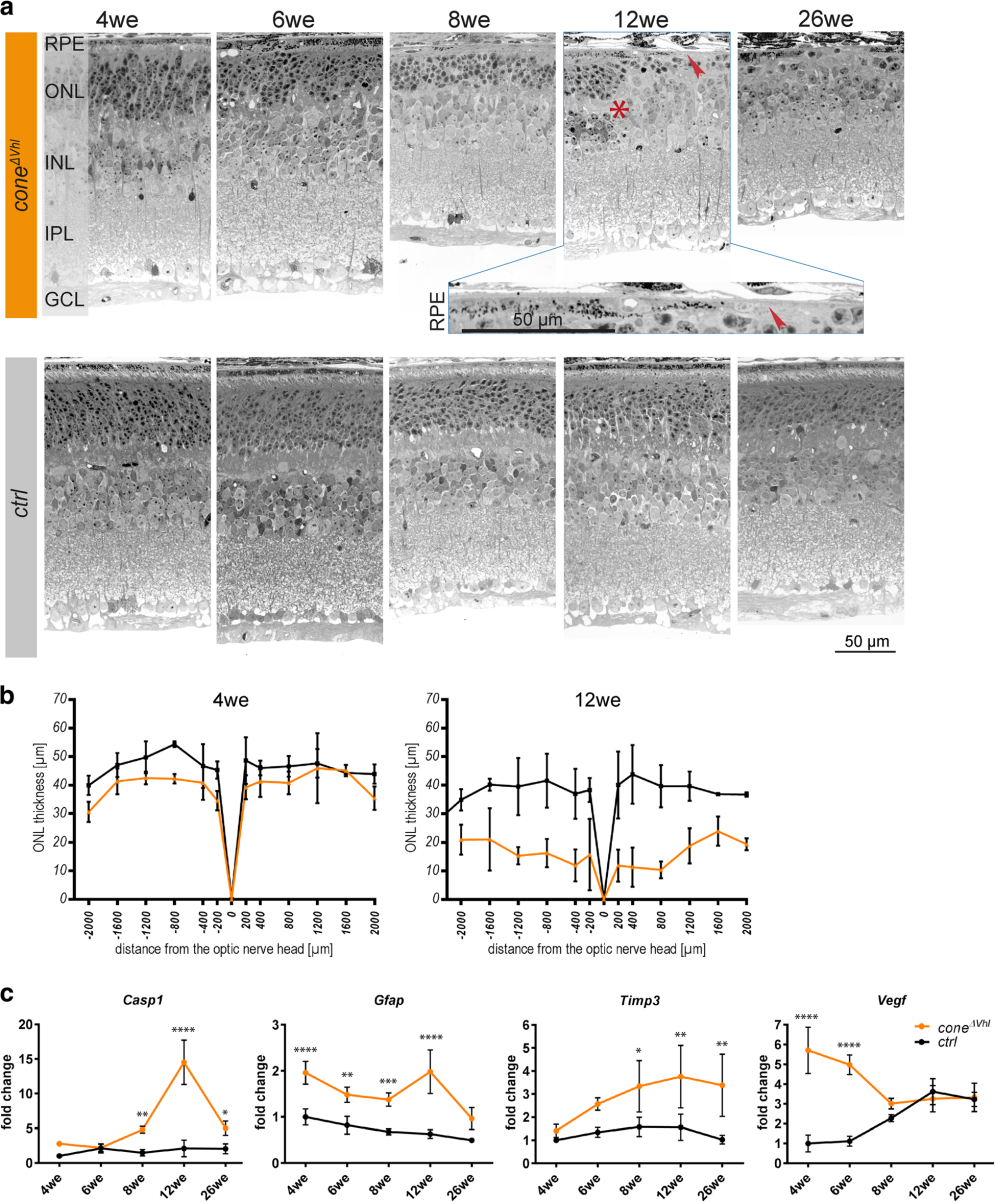
Progressive cone degeneration in *cone*^*ΔVhl*^ mice. **a** Inactivation of *Vhl* in cones led to progressive retinal degeneration (upper panels) as compared to *ctrl* mice (lower panels). *Cone*^*ΔVhl*^ mice displayed disturbed layering with reorganization of the INL (asterisk, 12we *cone*^*ΔVhl*^), severe loss of the ONL and partial loss of the RPE (arrowhead, 12we *cone*^*ΔVhl*^, inset) at 12 and 26 weeks of age. Abbreviations as in Fig. 1. Scale bar 50 μm. **b** Line chart of ONL thickness in *cone*^*ΔVhl*^ mice (orange) as compared to *ctrl* mice (black) at 4 and 12 weeks of age. *n*≥3. **c** Relative expression levels of genes involved in stress signaling and degeneration. Expression levels were normalized to *Actb* and calculated relatively to 4-week-old *ctrl* mice, which were set to 1. Shown are means ± SD. Two-way ANOVA with Šídák’s multiple comparison test was used for statistical analysis (*Casp1*: **p=0.0026, ****p<0.0001, *p=0.0244, *Gfap*: ****p<0.0001, **p=0.0035, ***p=0.0004, ****p<0.0001, *Timp3*: *p=0.0235, **p=0.0036, **p=0.0035, *Vegf*: ****p<0.0001, *n*≥3 per time point). *Casp1*: caspase 1, *Gfap*: glial fibrillary acidic protein, *Timp3*: tissue inhibitor of metalloproteinase 3, *Vegf*: vascular endothelial growth factor

We observed an age-dependent decline in HIF1A protein levels and hypoxia target gene expression in *cone*^*ΔVhl*^ mice (Fig. 1c, d). This prompted us to analyze retinal function and morphology. B-wave amplitudes and photopic ERGs were not significantly different between *cone*^*ΔVhl*^ mice and *ctrl* mice at 6 weeks of age (Fig. 2a). However, the amplitudes were strongly reduced in 12-week-old *cone*^*ΔVhl*^ mice as compared to age-matched *ctrl* mice (Fig. 2b). This indicated an age-dependent loss of function potentially due to retinal degeneration. Indeed, we observed severe, progressive thinning of the outer nuclear layer (ONL) in *cone*^*ΔVhl*^ mice with most cones lost at 26 weeks of age (Fig. 3a, b). Additionally, partial loss of the retinal pigment epithelium (RPE) and strong perturbations in the inner nuclear layer (INL) were detected in older *cone*^*ΔVhl*^ mice (Fig. 3a, 12we *cone*^*ΔVhl*^). In contrast, the *ctrl* mice showed only a slow age-related ONL thinning, as reported for the parental *R91W;Nrl*^*-/-*^ mouse line before [32].

To gain insight into cellular mechanisms leading to cone degeneration, we analyzed expression of specific genes during aging in *cone*^*ΔVhl*^ and *ctrl* mice (Fig. 3c). Caspase 1 (*Casp1*), a gene involved in retinal degeneration, was upregulated in *cone*^*ΔVhl*^ mice at 8 weeks and peaked at 12 weeks of age. Expression of the stress signaling gene glial fibrillary acidic protein (*Gfap*) was upregulated in *cone*^*ΔVhl*^ mice already at 4 weeks and remained elevated throughout the observation period. Interestingly, we observed increased expression of tissue inhibitor of metalloproteinase 3 (*Timp3*) during the aging process, a gene associated with age-related macular degeneration [43, 44]. Similarly, expression of vascular endothelial growth factor (*Vegf*), a hypoxic response gene that is involved in neovascularization in wet AMD, was strongly increased in *cone*^*ΔVhl*^ mice at 4 and 6 weeks of age.

### Subretinal neovascularization and vascular leakage in *cone*^*ΔVhl*^ mice

**Fig. 4:**
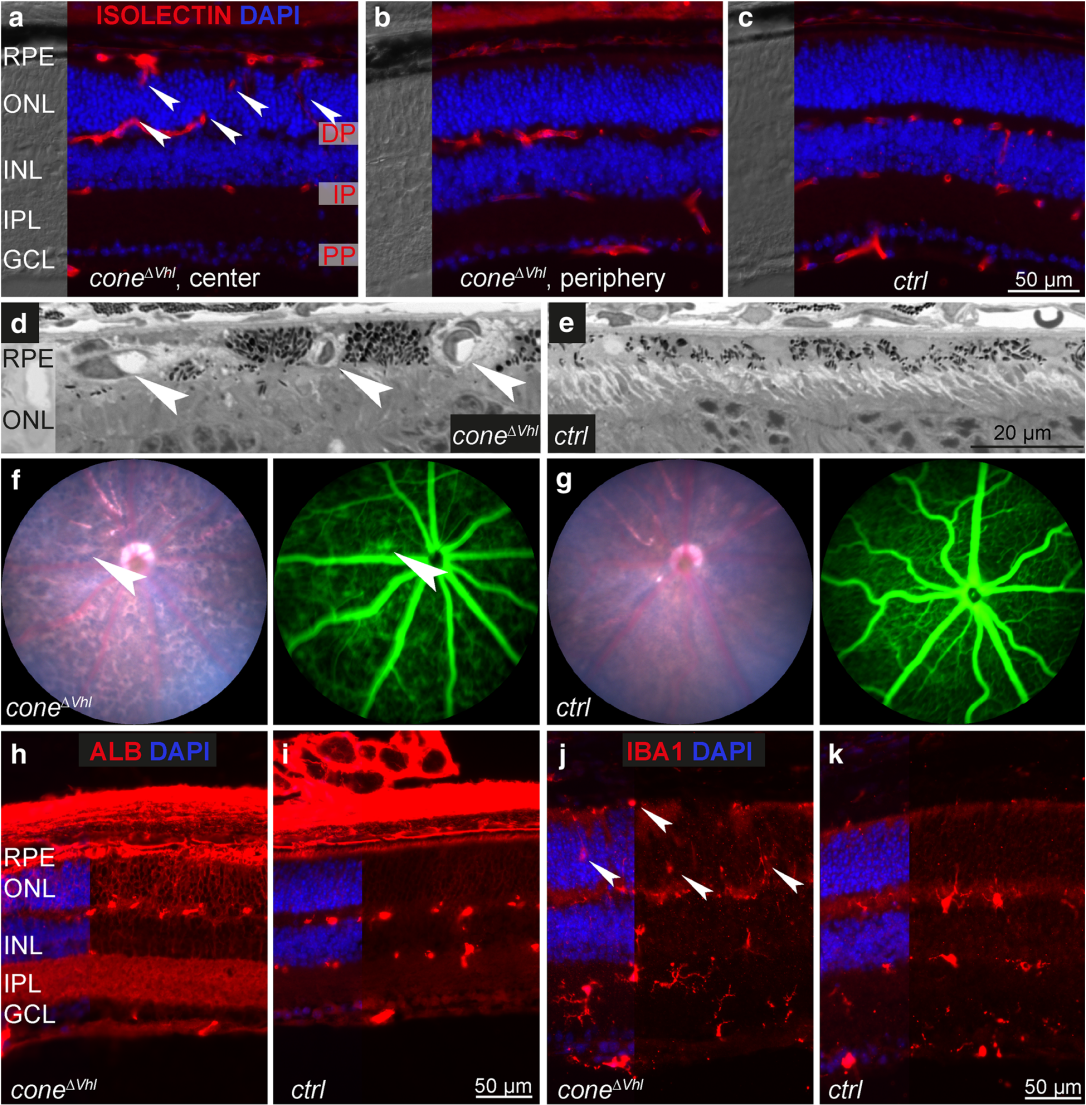
Subretinal neovascularization in *cone*^*ΔVhl*^ mice. **a-c** Immunostaining of cross-sections of the central (**a**) and peripheral (**b**) retina of 4-week-old *cone*^*ΔVhl*^ and age-matched *ctrl* mice (**c**, central retina) with isolectin. Arrowheads indicate vessels extending from the deep plexus into the ONL in central retinas of *cone*^*ΔVhl*^ mice. DP: deep plexus, IP: intermediate plexus, PP: primary plexus. Scale bar 50 μm. **d, e** Light microscopy of 4-week-old *cone*^*ΔVhl*^ and *ctrl* mice. Neovessels (arrowheads) reached the RPE, but did not cross Bruch’s membrane. Scale bar 20 μm. **f, g** Fundus imaging and fluorescein angiography of *cone*^*ΔVhl*^ mice and *ctrl* mice at 4 weeks of age. Signs of leakage (arrowhead) were detected in *cone*^*ΔVhl*^ mice. **h-k** Retinal cross-sections of *ctrl* **h, j** and *cone*^*ΔVhl*^ mice (**i, k**) were stained with antibodies against ALB (**h, i**) and IBA1 (**j, k**) at 4 weeks of age. Microglia/macrophages were found within the photoreceptor layer and RPE in *cone*^*ΔVhl*^ mice (arrowheads). Scale bar 50 μm. Abbreviations as in Fig. 1

Increased transcription of *Timp3* is potentially associated with a higher risk to develop neovascular AMD [43] and hypoxia-regulated genes such as *Vegf* contribute to retinal and choroidal neovascularization (reviewed in [45]). Therefore, we analyzed the vascular network of *cone*^*ΔVhl*^ mice. All three vascular plexi were detected at 4 weeks of age (Fig. 4a, b, c). However, we observed vessels extending from the deep plexus into the normally avascular ONL in central retinas of *cone*^*ΔVhl*^ mice (Fig. 4a, arrowheads). Vessels reached the RPE but did not cross Bruch’s membrane (Fig. 4d, e), as we never observed retinal-choroidal anastomoses. This suggests that abnormal vessels originated from the retinal vasculature and not from the choroid. Interestingly, abnormal vessel growth was characteristic for the central but not for the peripheral retina (Fig. 4b). To evaluate the integrity of retinal vessels we performed fluorescein angiography. Signs of leakage were observed in *cone*^*ΔVhl*^ mice (Fig. 4f, g). To confirm these findings we stained retinal cross-sections for albumin (ALB), a marker for blood extravasation. Detailed analysis revealed strong ALB immunoreactivity in the ONL, RPE and inner plexiform layer (IPL) in *cone*^*ΔVhl*^ mice, as opposed to the signal detected in *ctrl* mice that was confined to retinal vessels. The choroid with its extensive vasculature was also strongly positive for ALB in both types of mice (Fig. 4h, i). Microglia/macrophages detected by IBA1 staining were found within the photoreceptor and subretinal layer in *cone*^*ΔVhl*^ but not *ctrl* mice (Fig. 4j, k).

### Early vascular defects in *cone*^*ΔVhl*^ mice

**Fig. 5:**
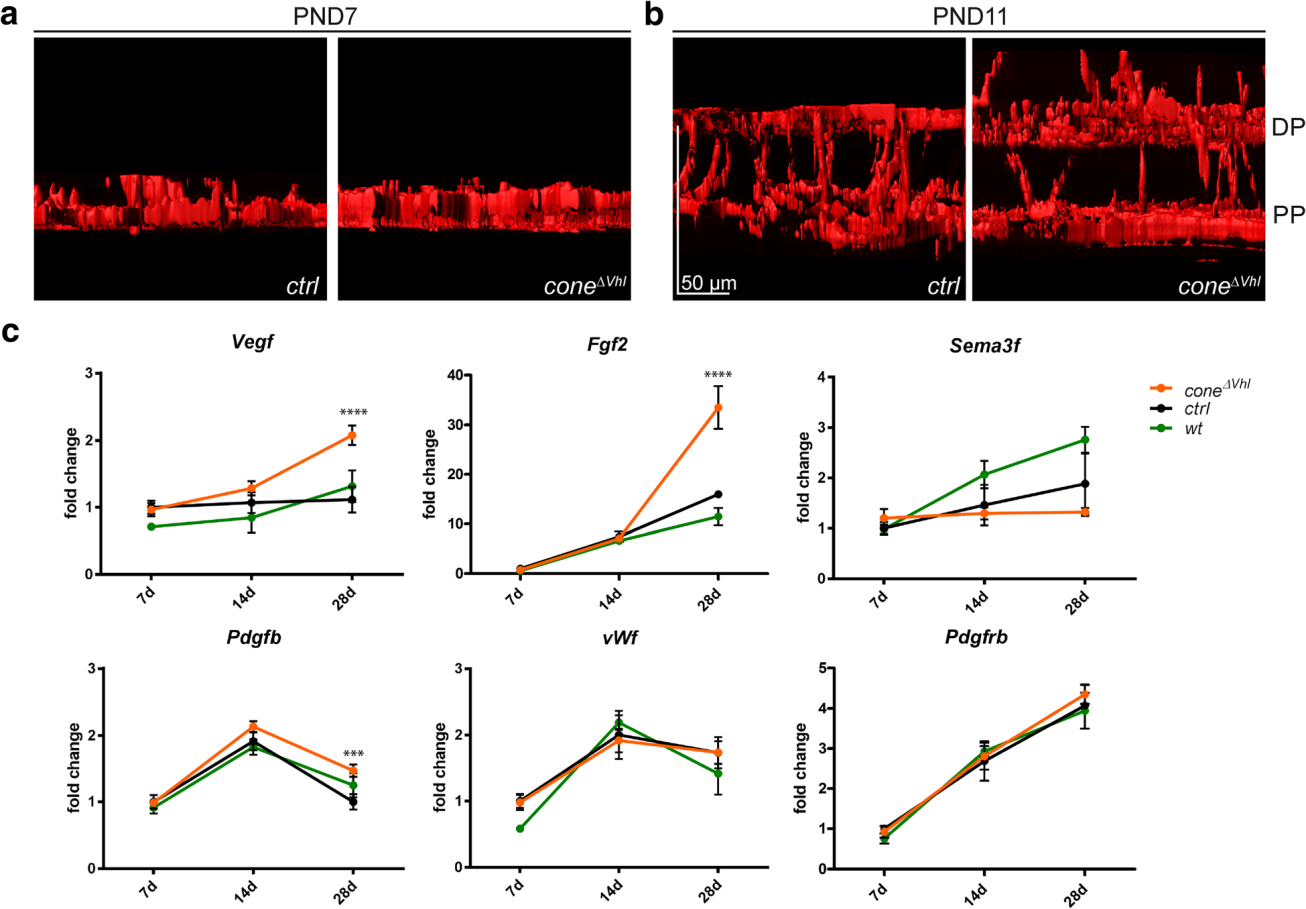
Pathological vessel growth starts around PND11 in *cone*^*ΔVhl*^ mice. **a, b** 3D-reconstruction of blood vessels stained with isolectin on retinal flat mounts of *ctrl* (left) and *cone*^*ΔVhl*^ (right) mice at PND7 (**a**) and PND 11 (**b**). At PND11, vessels extended from the deep plexus towards the ONL. The intermediate plexus has not yet been formed in both *ctrl* and *cone*^*ΔVhl*^ mice. For better recognition and distinction the z-value of the z-stacks was increased five times. DP: deep plexus, PP: primary plexus. Scale bar 50 μm. **c** Relative expression levels of angiogenic genes in retinas of *cone*^*ΔVhl*^ (orange), *ctrl* (black) and *wt* (green) mice at indicated time points. Expression levels were normalized to *Actb* and compared to *ctrl* mice at PND7, which were set to 1. Shown are means ± SD. Two-way ANOVA with Šídák’s multiple comparison test was used for statistical analysis comparing *ctrl* with *cone*^*ΔVhl*^ mice (*Vegf*: ****p<0.0001, *Fgf2*: ****p<0.0001, *Pdgfb*: ***p=0.0009, *n*=3 per time point). *Vegf*: vascular endothelial growth factor, *Fgf2*: fibroblast growth factor 2, *Sema3f*: semaphorin 3F, *Pdgfb*: platelet derived growth factor, B polypeptide, *vWf*: von Willebrand factor*, Pdgfrb*: platelet derived growth factor receptor, beta polypeptide

S-opsin expression starts shortly before birth in mice [46, 47] and we observed *Cre*-activity in *BPCre;R91W;Nrl*^*-/-*^*;ZsGreen* reporter mice as early as at postnatal day (PND) 1 (data not shown). The primary plexus in mice develops along a central-to-peripheral gradient and reaches the periphery around PND8-10 [48]. Vessels sprout from the primary plexus into the retina and turn laterally when they reach the outer and inner boundaries of the INL to first form the deep plexus and subsequently the intermediate plexus [48]. To determine the onset of neovascularization in *cone*^*ΔVhl*^ mice we stained retinal flat mounts with isolectin at PND7 and PND11. Using 3D-reconstruction of blood vessels, no difference in early vessel formation was detected between *cone*^*ΔVhl*^ and *ctrl* mice at PND7 (Fig. 5a). However, at PND11, before formation of the intermediate plexus, vessels growing from the deep plexus into the ONL were observed in *cone*^*ΔVhl*^ mice (Fig. 5b).

Formation of the deep plexus is preceded by *Vegf* expression in the INL [49]. We analyzed gene expression levels in *cone*^*ΔVhl*^, *ctrl* and *129S6* (*wt,* rod-dominant retina) mice to test for different regulation of genes involved in angiogenesis and vessel guidance during and after the process of vessel formation (PND7, 14 and 28, Fig. 5c). *Vegf* mRNA was not elevated at PND7, slightly increased by 1.3-fold at PND14 and significantly increased at PND28 (2-fold) in *cone*^*ΔVhl*^ mice compared to controls. Fibroblast growth factor 2 (*Fgf2*), a potent angiogenic factor that has been shown to be involved in retinal stress [50–52], was upregulated at PND28, at a time when developmental vascularization is completed. Recently, protective effects of semaphorin 3F (*Sema3f*) against subretinal neovascularization have been demonstrated [53]. We thus analyzed *Sema3f* gene expression levels to test for potential differential expression of this anti-angiogenic factor in *cone*^*ΔVhl*^ mice. However, expression of *Sema3f* in all-cone mice did not differ between *ctrl* and *cone*^*ΔVhl*^ mice (Fig. 5c). On the other hand, a pro-angiogenic factor, platelet derived growth factor, B polypeptide (*Pdgfb*), was upregulated in *cone*^*ΔVhl*^ compared to *ctrl* mice at PND28. *Pdgfb* expression by endothelial cells is essential for pericyte recruitment and is increased under hypoxia [54–57]. Surprisingly, expression levels of von Willebrand factor (*vWf*), a marker for endothelial cells, as well as of *Pdgfb receptor* (*Pdgfrb*), which is expressed by pericytes/mural cells [58–61], were not increased in *cone*^*ΔVhl*^ mice as compared to controls. The reason for this unexpected pattern of expression is not clear and requires further investigation.

### HIF1 is responsible for pathological vessel growth and progressive cone degeneration in *cone*^*ΔVhl*^ mice

**Fig. 6:**
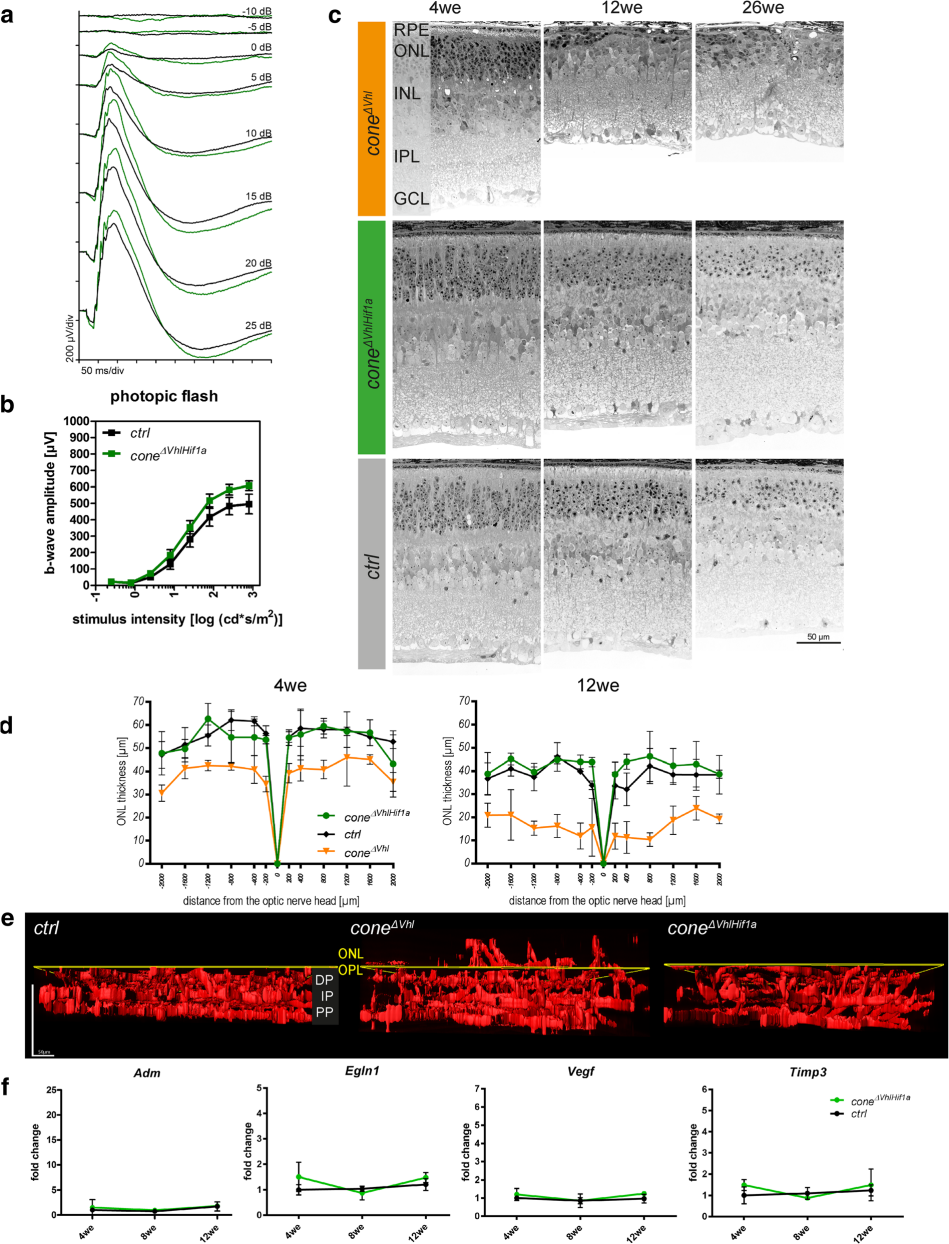
Combined deletion of *Hif1a* and *Vhl* (*cone*^*∆VhlHif1a*^) rescues the defects in retinal function, morphology and vasculature observed in *cone*^*∆Vhl*^ mice. **a, b** Average single-flash photopic ERGs and b-wave amplitudes showed no significant differences in *cone*^*∆VhlHif1a*^ mice (green) compared to *ctrl* mice (*R91W;Nrl*^*-/-*^*;Vhl*^*f/f*^; *Hif1a*^*f/f*^, black) at 12 weeks of age. *n*=4 per group. b-wave amplitudes are shown as means ± SD. **c, d** Light microscopy analysis and measurement of the ONL thickness in *cone*^*∆VhlHif1a*^ (green), *ctrl* mice (*R91W;Nrl*^*-/-*^*;Vhl*^*f/f*^; *Hif1a*^*f/f*^, black) and *cone*^*ΔVhl*^ mice (orange, as shown in Fig. 3b) at indicated time points. Abbreviations as in Fig. 1. Scale bar 50 μm. **e** 3D-reconstruction of blood vessels stained with isolectin on retinal flat mounts of *ctrl* (left), *cone*^*ΔVhl*^ (middle) and *cone*^*∆VhlHif1a*^ mice (right). Inactivation of *Hif1a* (*cone*^*∆VhlHif1a*^) rescued the pathological neovascularization. Analysis at 4 weeks of age, *n*=3. For better recognition and distinction the z-value of the z-stacks was increased five times. The yellow line indicates the border between the outer plexiform layer (OPL) and the ONL. DP: deep plexus, IP: intermediate plexus, PP: primary plexus. **f** Relative gene expression of hypoxic target genes in *cone*^*∆VhlHif1a*^ mice (green) and *ctrl* mice (*R91W;Nrl*^*-/-*^*;Vhl*^*f/f*^; *Hif1a*^*f/f*^, black) at 4we, 8we and 12we as indicated. Abbreviations as in Figs. 1 and 3. Expression levels were normalized to *Actb* and compared to 4-week-old *ctrl* mice, which were set to 1. Shown are means ± SD. *n*≥3 per time point. Scale bar 50 μm

We hypothesized that stabilized HIF1A, and not other *Vhl* targets, might promote retinal degeneration and neovascularization in *cone*^*ΔVhl*^ mice. Therefore, we additionally deleted *Hif1a* in cone photoreceptors (*BPCre;R91W;Nrl*^*-/-*^*;Vhl*^*f/f*^*;Hif1a*^*f/f*^*=cone*^*ΔVhlHif1a*^). After we confirmed the presence of deletion alleles for both *Hif1a* and *Vhl* in genomic DNA of *cone*^*ΔVhlHif1a*^ retinas (not shown) we determined retinal function. At 12 weeks of age, photopic ERG traces and b-wave amplitudes were similar in *cone*^*ΔVhlHif1a*^ and *ctrl* (*R91W;Nrl*^*-/-*^*; Vhl*^*f/f*^*;Hif1a*^*f/f*^) mice (Fig. 6a, b) indicating no functional loss upon combined *Hif1a* and *Vhl* inactivation. Similarly, retinal morphology (Fig. 6c) and ONL thickness (Fig. 6d) of *cone*^*ΔVhlHif1a*^ mice was comparable to *ctrl* mice, whereas the thickness of the ONL in *cone*^*ΔVhl*^ mice was prominently reduced (same data as in Fig. 3b) at 12 weeks of age.

Combined deletion of *Hif1a* and *Vhl* also prevented pathological neovascularization into the ONL but had no effect on the developmental formation of the three vascular plexi (Fig. 6e). No induction of the expression of hypoxic target genes such as *Adm*, *Egln1*, *Vegf* and *Timp3* was detected in *cone*^*ΔVhlHif1a*^ mice, confirming that HIF1 was the responsible HIF isoform for regulating their expression in cones (Fig. 6f). Altogether, these data demonstrate that combined deletion of *Hif1a* and *Vhl* (*cone*^*ΔVhlHif1a*^ mice) rescues the retinal phenotype observed in *cone*^*ΔVhl*^ mice and identify HIF1 as the causative factor for retinal degeneration and pathological vessel growth in *cone*^*ΔVhl*^ mice.

## Discussion

Previously, it has been demonstrated that the knockdown of *Vhl* in rod cells causes HIF1A stabilization under normoxic conditions [39]. Here, we particularly investigated the effects of a chronic hypoxia-like response in cone photoreceptors. We show that ablation of *Vhl* in cones resulted in a chronic hypoxia-like situation with accumulation of HIF1A and induction of HIF1A target genes. This led to pathological vessel growth into the photoreceptor layer, reduced retinal function and severe, progressive cone degeneration. These consequences resemble some of the features of AMD pathology, particularly of a subset of neovascular AMD known as RAP. In human patients, HIF1A and HIF2A were detected in macrophages and endothelial cells of neovascular membranes associated with AMD [62]. Additionally, the correlation of drusen density and decreased choroidal blood flow [26] as well as choroidal ischemia in AMD patients [23–25] suggest reduced oxygen transport from the choroid to the inner retina [63]. Therefore, tissue hypoxia and HIFs might play an important role in disease development and/or progression.

Expression of numerous genes involved in metabolism, stress, cell survival and angiogenesis was increased in *cone*^*∆Vhl*^ mice (Fig. 1d, 3c, 5c), as was *Timp3* (Fig. 3c). TIMP3 is an important regulator of extracellular matrix (ECM) remodeling through inhibition of matrix metalloproteases and has been suggested to be a senescence-related protein [64] that is potentially regulated by HIF2 in rods [38]. Importantly, TIMP3 inhibits angiogenesis by interacting with VEGF [65] and is relevant for Bruch’s membrane integrity [64, 66]. Furthermore, mutations in *Timp3* have been associated with Sorsby’s fundus dystrophy, an autosomal dominant maculopathy with submacular choroidal neovascularization [67, 68]. We can only speculate about the reasons for elevated *Timp3* during the aging process in *cone*^*∆Vhl*^ mice but it seems plausible that retinas of *cone*^*∆Vhl*^ mice require extensive ECM remodeling due to progressive cone degeneration (Fig. 3).

We detected early and prominent vascular defects with abnormal vessel growth into the ONL, subretinal space and RPE layer in *cone*^*∆Vhl*^ mice. Increased VEGF levels in transgenic mice expressing *Vegf* under the transcriptional control of the rhodopsin promoter (*rho/VEGF* mice) were shown to cause subretinal neovascularization [69–71]. In our model, *Vegf* was not upregulated at PND14, at a time when vessels had already started growing into the ONL (Fig. 5). However, transient *Vegf* expression in the INL regulates the formation of the deep plexus during development [49], i.e. already minor changes in the local *Vegf* concentration gradient, which are not detectable by RT-PCR, may misguide retinal vessels in *cone*^*∆Vhl*^ mice. To date, it remains unclear which pro-angiogenic factor(s) are responsible for the observed neovascularization. Nonetheless, our findings demonstrate that this is a *Hif1a*-mediated mechanism, as additional deletion of *Hif1a* fully rescued the vascular defects (Fig. 6).

Interestingly, cone-specific deletion of *Hif1a* did not affect formation of the three vascular plexi (Fig. 6e), while it has been shown that knockdown of *Hif1a* in most cells of the retinal periphery prevented formation of the intermediate plexus [42]. Thus, our results suggest that *Hif1a* expression in cones, as opposed to other cells presumably in the INL, is not essential for the development of the intermediate plexus.

Our data do not define whether cone degeneration in *cone*^*∆Vhl*^ mice is due to the effects of the chronic intrinsic activation of the hypoxic response or to early development of vascular defects. Another mouse model shows, however, that a chronic hypoxia-like response in rods leads to slow photoreceptor degeneration in the absence of reported vascular defects (*rod*^*∆Vhl*^ mice [39]). This suggests that the long-term activation of HIF1 may reduce cell survival by an intrinsic mechanism. During hypoxia, HIF1 regulates mitochondrial respiration and thus metabolic adaptation towards glycolysis [72] leading to reduced energy (ATP) levels and potentially starvation. It has been shown that starvation of cones may lead to cone cell death [73]. Furthermore, it has been suggested that cones might be more sensitive to reduced oxygen and nutrient levels compared to rods, for reasons not fully understood [74, 75]. This could possibly explain the progressive degeneration and the accelerated phenotype in *cone*^*∆Vhl*^ compared to *rod*^*∆Vhl*^ mice.

It might also be possible, however, that photoreceptor degeneration is secondary to the early pathological vascular defects. Inactivation of *Vhl* in the retinal periphery during development leads to vessel growth into the ONL and severe retinal degeneration [52, 76]. Whereas the retinal periphery was strongly degenerated at 10 weeks of age, photoreceptor loss in the normal vascularized central retina was less pronounced [52]. We observed microglia activation and impaired function of the blood-retina-barrier in *cone*^*∆Vhl*^ mice, as shown by IBA1 and ALB staining, respectively (Fig. 4h-k). It seems likely that retinal hemorrhages exacerbate cone photoreceptor degeneration in our model, as suggested for other models [77, 78]. Photoreceptor degeneration was also observed in very low density lipoprotein receptor knockout mice (*Vldlr*^*-/-*^; [79]) where early retinal neovascularization causes vessels to extend from the deep plexus into the subretinal space [80, 81]. Interestingly, it has been suggested that the *Vldlr*^*-/-*^ phenotype is linked to HIF1A. Joyal and colleagues proposed that starved *Vldlr*^*-/-*^ photoreceptors have reduced amounts of the Krebs cycle metabolite α-ketoglutarate, which decreases PHD activity and thus promotes stabilization of HIF1A. Consequently, *Vegf* is secreted, leading to RAP-like neovascularization [82]. We hypothesize that the combination of early vascular defects and the activation of a chronic hypoxia-like response, which affects expression of genes involved in cellular metabolism and retinal stress, leads to the progressive cone degeneration observed in *cone*^*ΔVhl*^ mice.

Our findings identify HIF1 in cones as the factor causing cone degeneration, pathological neovascularization and loss of function. Similarly, chronic activation of the hypoxic response in rods resulted in an HIF1- and age-dependent retinal degeneration (Barben et al., submitted). Whereas this provides strong evidence that activation of HIF1 in rod and cone photoreceptors leads to retinal degeneration, the activation of HIF2 but not of HIF1 is responsible for metabolic stress in RPE upon RPE-specific *Vhl* inactivation [75]. Thus, our data and data published by others suggest cell type-specific roles for HIF1A and HIF2A in retinal pathology.

## Conclusions

Our data demonstrate that a chronic hypoxic response in cone photoreceptors induces HIF1A-mediated pathological vessel growth and cone degeneration. Evidence shows that HIF1A can be safely inactivated in cones (see above), rods ([38], Barben et al., submitted) and RPE [75] suggesting that targeting HIF transcription factors in photoreceptors and RPE may provide a potential therapeutic approach to rescue hypoxia-mediated retinal degeneration in patients and an alternative to anti-VEGF agents.

## Abbreviations

*Adm*: Adrenomedullin
ALB: Albumin
AMD: Age-related macular degeneration
*Bnip3*: BCL2/adenovirus E1B 19-kDa interacting protein 3
*Casp1*: Caspase 1
CNV: Choroidal neovascularization
DAPI: 4',6-Diamidine-2'-phenylindole dihydrochloride
ECM: Extracellular matrix
*Egln1*: Egl-9 family hypoxia-inducible factor 1 (known as *Phd2*)
ERG: Electroretinography
*Fgf2*: Fibroblast growth factor 2
*Gfap*: Glial fibrillary acidic protein
*Glut1*: Glucose transporter 1
HIF1A: Hypoxia-inducible factor 1A
IBA1: Alias, allograft inflammatory factor 1
INL: Inner nuclear layer
IPL: Inner plexiform layer
ONL: Outer nuclear layer
*Pdgfb*: Platelet derived growth factor, B polypeptide
*Pdgfrb*: Pdgfb receptor
PFA: Paraformaldehyde
PHD: Prolyl hydroxylase domain
PND: Postnatal day
RAP: Retinal angiomatous proliferation
RPE: Retinal pigment epithelium
RT: Room temperature
*Sema3f*: Semaphorin 3F
*Timp3*: Tissue inhibitor of metalloproteinase 3
VEGF: Vascular endothelial growth factor
VHL: Von Hippel Lindau protein
*Vldlr*: Very low density lipoprotein receptor
*vWf*: Von Willebrand factor
